# RNA-Seq Based Transcriptome Analysis of the Type I Interferon Host Response upon Vaccinia Virus Infection of Mouse Cells

**DOI:** 10.1155/2017/5157626

**Published:** 2017-02-09

**Authors:** Bruno Hernáez, Graciela Alonso, Juan Manuel Alonso-Lobo, Alberto Rastrojo, Cornelius Fischer, Sascha Sauer, Begoña Aguado, Antonio Alcamí

**Affiliations:** ^1^Centro de Biología Molecular Severo Ochoa, Consejo Superior de Investigaciones Científicas-Universidad Autónoma de Madrid (CSIC-UAM), 28049 Madrid, Spain; ^2^Max Planck Institute for Molecular Genetics, 14195 Berlin, Germany; ^3^Max Delbrück Center for Molecular Medicine, Robert-Rössle-Str. 10, 13092 Berlin, Germany

## Abstract

Vaccinia virus (VACV) encodes the soluble type I interferon (IFN) binding protein B18 that is secreted from infected cells and also attaches to the cell surface, as an immunomodulatory strategy to inhibit the host IFN response. By using next generation sequencing technologies, we performed a detailed RNA-seq study to dissect at the transcriptional level the modulation of the IFN based host response by VACV and B18. Transcriptome profiling of L929 cells after incubation with purified recombinant B18 protein showed that attachment of B18 to the cell surface does not trigger cell signalling leading to transcriptional activation. Consistent with its ability to bind type I IFN, B18 completely inhibited the IFN-mediated modulation of host gene expression. Addition of UV-inactivated virus particles to cell cultures altered the expression of a set of 53 cellular genes, including genes involved in innate immunity. Differential gene expression analyses of cells infected with replication competent VACV identified the activation of a broad range of host genes involved in multiple cellular pathways. Interestingly, we did not detect an IFN-mediated response among the transcriptional changes induced by VACV, even after the addition of IFN to cells infected with a mutant VACV lacking B18. This is consistent with additional viral mechanisms acting at different levels to block IFN responses during VACV infection.

## 1. Introduction

Type I interferons (IFNs) constitute a family of related cytokines (IFN-*α* subtypes, IFN-*β*, and other IFN family members) that bind a common and heterodimeric cell surface receptor (IFNAR) and play an important role in the first line of defence against virus infections [1–3]. After initial molecular recognition of the invading virus by host cell pattern recognition receptors (PRRs), these IFNs are secreted and bind cognate cellular receptors to exert their function either locally or distally. This binding initiates the Janus kinase (JAK)/signal transducers and activators of transcription (STAT) signalling cascade to trigger the activation of diverse host genes, depending on cell type, with potent antiviral activity that contributes to the establishment of an antiviral state in the adjacent healthy cells and the activation of the apoptotic program to eliminate infected cells. Thus, the main purpose of the IFN response is to limit virus replication and infection spreading [4].

Vaccinia virus (VACV) is the most studied member of the Poxviridae family of large DNA viruses with cytoplasmic replication. VACV is the vaccine used to eradicate smallpox more than 30 years ago and constitutes an excellent model to analyze the evasion of the IFN based host response to viral infection. Viruses have to neutralize the antiviral activity of IFNs, and in this sense VACV and other poxviruses seem to be unique encoding a plethora of genes to this effect (reviewed in [2, 3, 5, 6]). Among others, VACV encodes the A46 and A52 protein to inhibit toll-like receptor (TLR) signalling that leads to IFN production [7] and VH1 to dephosphorylate STAT1 and STAT2 [8, 9] but also diverse proteins to specifically inhibit the antiviral activity of some IFN-induced genes. This is the case of the E3 and K3 proteins that employ two different mechanisms to counteract double-stranded RNA-dependent protein kinase (PKR) effector functions [10, 11]. Additionally, E3 binds the product of the IFN-stimulated gene 15 (ISG15) to prevent its antiviral action [12]. But one the most efficient strategies employed by poxviruses to avoid IFN effects is to encode IFN binding proteins that are secreted from infected cells to prevent the interaction of IFNs with their cellular receptors. In the case of VACV strain Western Reserve (WR), the type I IFN binding protein is encoded by the* B18R* gene (*B19R* in the Copenhagen strain). A relevant role of this protein in VACV pathogenesis was soon assigned, since the lack of* B18R* expression after intranasal infection of mice resulted in an attenuated virus, indicating that blocking the IFN host response is crucial for the development of VACV infection [13]. The B18 protein has no amino acid sequence similarity to cellular IFN receptors and, in contrast to the cellular counterparts, binds IFN*α*/*β* from a broad range of host species [13]. The protein is synthesized early after VACV infection, is secreted into the medium, and is found as a soluble form or anchored to the cell surface [14, 15]. This binding to the cell surface has been shown to occur* via* interaction of the B18 amino terminus with glycosaminoglycans (GAGs) [16] and allows B18 to prevent the establishment of an IFN-induced antiviral state in cells surrounding the infection site.

In the present study, by using RNA sequencing with the Illumina technology (RNA-seq) and differential gene expression analyses, we have further analyzed the ability of B18 to block the IFN based response in a mouse fibroblast cell line. We also extend the study to VACV-infected cells to identify changes in host gene expression profile induced by VACV or a VACV mutant lacking the* B18R* gene (VACVΔB18), with special emphasis on the inhibition of the type I IFN-induced host cell response.

## 2. Materials and Methods

### 2.1. Cell Culture and Reagents

Mouse L929 cells were used to obtain RNA samples for high-throughput sequencing, while BSC-1 cells (African green monkey kidney origin) were used to prepare virus stocks. Recombinant His-tagged VACV B18 protein was expressed in the baculovirus system and purified as previously described [17]. Protein purity was checked on Coomassie blue-stained SDS-PAGE and quantified by gel densitometry. Murine recombinant IFN-*α* subtype A was purchased from PBL Assay Science (>95% pure), diluted in phosphate-buffered saline, and maintained at −70°C until use.

### 2.2. Viruses and Infections

Virulent VACV strain WR and the correspondent VACV mutant lacking B18R expression (VACVΔB18, [14]) were grown in BSC-1 cells and stocks of semipurified virus were prepared by sedimentation through a 36% sucrose cushion. L929 cells were infected with VACV or VACVΔB18 with a multiplicity of infection of 5 plaque forming units (pfu)/cell in order to ensure the infection of all cells to obtain a representative RNA-seq profile of each condition. After adsorption of virus for 1 h at 37°C, the virus-containing medium was removed, and cells were washed twice with phosphate-buffered saline and replaced with fresh culture medium supplemented with 2% fetal bovine serum. Infected cells were then incubated at 37°C and harvested at 4 or 8 h postinfection (hpi) by scrapping. Where indicated, IFN (50 units/ml) was added to the infected cultures at 4 hpi and the incubation extended at 37°C to 9 hpi. Inactivation of viruses was performed as previously described [18], by incubation with 2 *μ*g/ml psoralen (4-9-aminomethyl-trioxsalen; Sigma) for 10 min and then UV-irradiated for 10 min with 2.25 J/cm^2^ in a Stratalinker 1800. Complete inactivation (>10^8^-fold reduction in pfu) was confirmed by plaque assay in BSC-1 cells.

### 2.3. RNA Extraction and Illumina RNA-Seq Library Preparation

Immediately after harvesting the samples, total cellular RNA was isolated from 1.2 × 10^6^ L929 cells using SV Total RNA Isolation System (Promega). RNA samples were quantified on a spectrophotometer (NanoDrop ND-1000; Thermo Scientific) and quality-analyzed in an Agilent 2100 Bioanalyzer (Agilent Technologies, Santa Clara, CA, US). All samples exhibited a RNA integrity number (RIN) over 9. The sequencing libraries were generated with TruSeq RNA Sample Prep Kit v2 Set A (Illumina). Briefly, poly(A) containing mRNA molecules were purified in two rounds using oligo(dT) attached magnetic beads from 1 *µ*g of total RNA. After chemical fragmentation, mRNA fragments were reverse-transcribed and converted into double-stranded cDNA molecules. Following end-repair and dA-tailing, paired-end sequencing adaptors were ligated to the ends of the cDNA fragments using TruSeq PE Cluster Kit v3-cBot-HS (Illumina).

### 2.4. Deep Sequencing and Sequence Analysis

Libraries were sequenced using TruSeq SBS Kit v3-HS (Illumina) on an Illumina Hiseq 2000 machine at the Max Planck Institute for Molecular Genetics, Berlin. More than 10^8^ 100 nt paired-end reads were obtained from each sample and after quality assessment with package FastQC (http://www.bioinformatics.babraham.ac.uk/projects/fastqc/), the fastq files containing these reads were mapped to the mouse genome (build GRCm38 from* Mus musculus* C57BL/6J strain) together with the VACV WR genome (Genebank, AY243312.1) using Tophat v2.0.4 with default parameters [19]. Only those reads aligned against mouse genome were considered in a differential gene expression analysis with Cuffdiff (Cufflinks v2.1.0 software [19]). Since biological duplicates of samples from untreated cells were available, all comparisons were performed against this sample using the default mode of Cuffdiff, which is the most suitable for our kind of data. Pathway analysis of the significantly differentially expressed genes detected was performed using Ingenuity Pathway Analysis (IPA) software. Creation of proportional Venn diagrams and gene expression heatmaps were generated with the R “VennDiagram v1.6.9” and “Gplots” packages, respectively. The raw RNA-seq data has been deposited at the European Nucleotide Archive (ENA) under the project number PRJEB15047.

### 2.5. mRNA Expression by Real-Time-PCR (RT-PCR)

To evaluate the expression levels of selected genes by RT-PCR, 1 *µ*g of DNA-free total RNA isolated from L929 cells (three biological replicates per condition) was used for first strand cDNA synthesis with iScript cDNA Synthesis (BioRad) using oligo(dT) and random primers. Quantitative polymerase chain reaction (qPCR) analysis was performed using Fast SYBR Green PCR Master Mix (Applied Biosystem) with three technical replicates for each biological replicate, according to the manufacturer's recommendation in an ABI 7900 HT system (Applied Biosystem). Gene-specific qPCR primers were designed using primer3Plus (http://www.bioinformatics.nl/cgi-bin/primer3plus/primer3plus.cgi/) and described in Table S1 in Supplementary Material available online at https://doi.org/10.1155/2017/5157626. Amplification was real-time-monitored and allowed to proceed in the exponential phase, until fluorescent signal reached a significant value (Ct). The fold change was determined using the 2^−ΔΔC(t)^ method [20].

## 3. Results

### 3.1. The Type I IFN Cellular Response Is Inhibited in the Presence of the VACV B18 Protein

**Figure 1:**
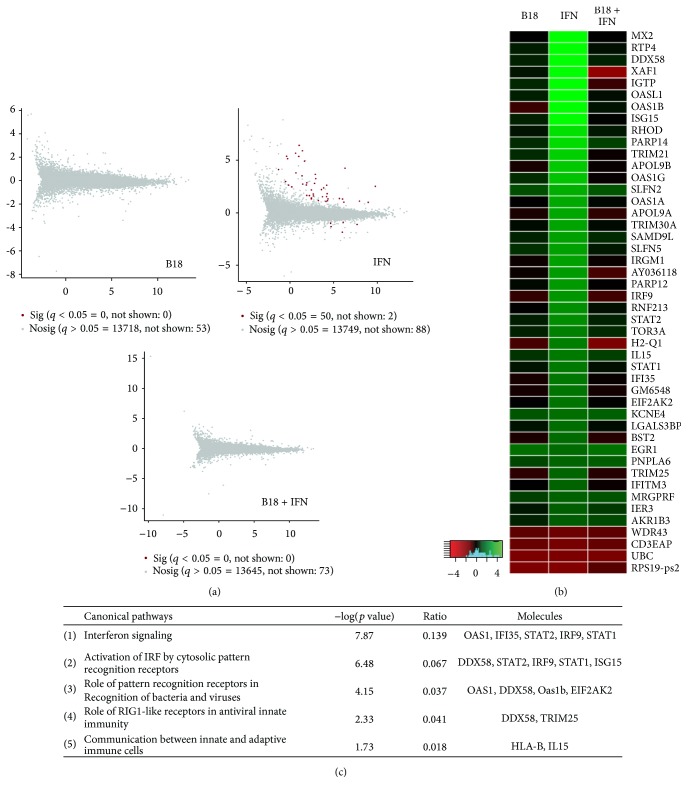
Effect of B18 on type I IFN response. L929 cells were incubated with recombinant B18 protein (B18), with mouse IFN*α* (IFN), or with recombinant B18 and then mouse IFN*α* (B18 + IFN), and analyzed by RNA-seq. (a) Corresponding *M*/*A* plots representing expression of all cellular genes. *M* value (log 2 fold change) from each transcript between untreated and indicated sample cells is plotted against *A* (overall average log expression level) of each untreated and indicated pair. Red dots indicate SDEGs when compared to untreated cells after differential expression analysis. (b) Heatmap of SDEGs identified after IFN treatment. The heatmap displays the fold change expression (log 2) in the indicated samples relative to results for untreated cells. The colour scale is shown at the bottom of the heatmap. (c) Enriched canonical pathways after IPA analysis with SDEGs identified after IFN induction.

To characterize the inhibitory role of the VACV type I IFN binding protein B18 on IFN signalling we analyzed the RNA-seq profile of mouse L929 cells incubated with recombinant B18 before and after IFN treatment. We first determined the effect of type I IFN on global cellular gene expression and performed high-throughput RNA sequencing on total RNA obtained from cells mock-treated or treated with 50 units/ml of IFN-*α* for 4 h. Under these conditions, we identified a set of 46 significantly differentially expressed genes (SDEGs) after IFN treatment when compared to mock-treated cells (Table S2). Most of them (42 genes) were found to be upregulated in response to IFN while only 4 genes were downregulated. This set of IFN-stimulated genes (ISGs) contained several genes with previously known direct antiviral activity, such as APOL9, BST2 (Tetherin), DDX58 (RIG-1), EIF2AK2 (PKR), IFITM3, ISG15, MX2, OAS-1, PARP12, or TRIM. We also identified some ISGs involved in the positive regulation of IFN production such as IRF9, STAT1, STAT2, TRIM21, or TRIM30 and others encoding immunomodulatory molecules such as IL15, H2-Q1 (HLA-B), or UBC. We considered this our high-confidence gene set and performed a pathway enrichment analysis that mainly identified IFN related canonical pathways as statistically significant enriched (Figure 1(c)), indicating that L929 cells used in this study exhibited an appropriate biological response to IFN.

However, when cells were incubated with 0.45 *µ*g/ml of recombinant protein B18 2 h before IFN addition we could not detect any significant change in cellular gene expression, indicating that the IFN-induced cell response was efficiently prevented by the addition of B18 (Figure 1). At the same time, it was confirmed that this concentration of B18 effectively protected against the antiviral effects of IFN (50 U/ml) using Vesicular stomatitis virus infection in HeLa cells (data not shown).

B18 is secreted from VACV-infected cells and has been previously shown to interact with GAGs at the surface of uninfected neighbouring cells to exert its inhibitory function [16]. This ability of B18 opens up the possibility of triggering additional signalling cascades after binding to GAGs on the cell surface. To test this possibility, cells were incubated for 4 h with the same amount of recombinant B18 used previously but in the absence of IFN. Importantly, under these conditions, no significant changes in the gene expression profile could be observed when compared to mock-treated cells, indicating no activation of host gene expression is triggered after the addition of B18 to cells (Figure 1).

**Figure 2:**
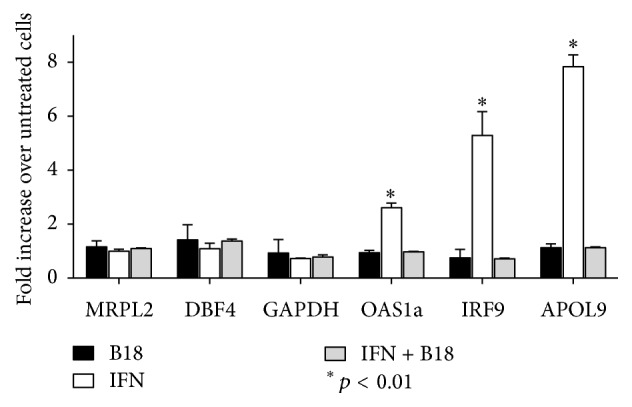
Confirmation of RNA-seq data by RT-qPCR after IFN induction. Gene expression for the indicated genes was assessed by RT-qPCR from L929 cells after incubation with B18, 50 units/ml IFN*α*, or IFN together with B18. *β*-Actin gene was used as a reference to normalize the data. Expression values are shown as fold change compared to untreated cells (mean ± SEM and significant differences are displayed).

To confirm these results, we selected three of the genes upregulated after IFN addition from the RNA-seq data (APOL9, IRF9, and OAS-1), together with other three genes whose expression was unaffected (DBF4, GAPDH, and MPRL2), and determined by RT-qPCR their expression levels. As expected, we found significant increased gene expression for APOL9, IRF9, and OAS-1 after IFN induction, and, concordant with the results from RNA-seq, the addition of B18 prior to IFN prevented this upregulation, keeping their expression values similar to those found in untreated cells (Figure 2). Moreover, DBF4, GAPDH, and MPRL2 expression determined by RT-qPCR remained unaffected after IFN induction or B18 incubation, as seen in the RNA-seq data (Figure 2).

### 3.2. VACV-Induced Changes on Cellular Gene Expression Profile

Searching for the initial response to VACV infection, we first explored by RNA-seq the transcriptomes of cells infected with UV-inactivated VACV and compared it with mock-treated cells. After alignment, only 0.17% of total reads matched the viral genome (Table S3), mostly corresponding to early VACV genes according to the temporal expression of VACV ORFs previously defined [21]. Under these conditions, we could identify changes in the expression of a modest set of 53 cellular genes (Table S4). Among these, the upregulation of some genes controlled by the NF-*κ*B complex such as CCL5 (RANTES), H2-Q1 (HLA-B), the protein phosphatase DUSP5 involved in negative regulation of MAP kinases, the transcription factor FOSB, or SERPINE1 and also the downregulation of the macrophage migration inhibitory factor (MIF), CXCL1, ABCG1, SOD3, and negative regulator of NF-*κ*B TRIB3 could represent the initial response to virus infection in the absence of viral genome replication. However the absence of type I IFN or IFN effectors should be noted.

**Figure 3:**
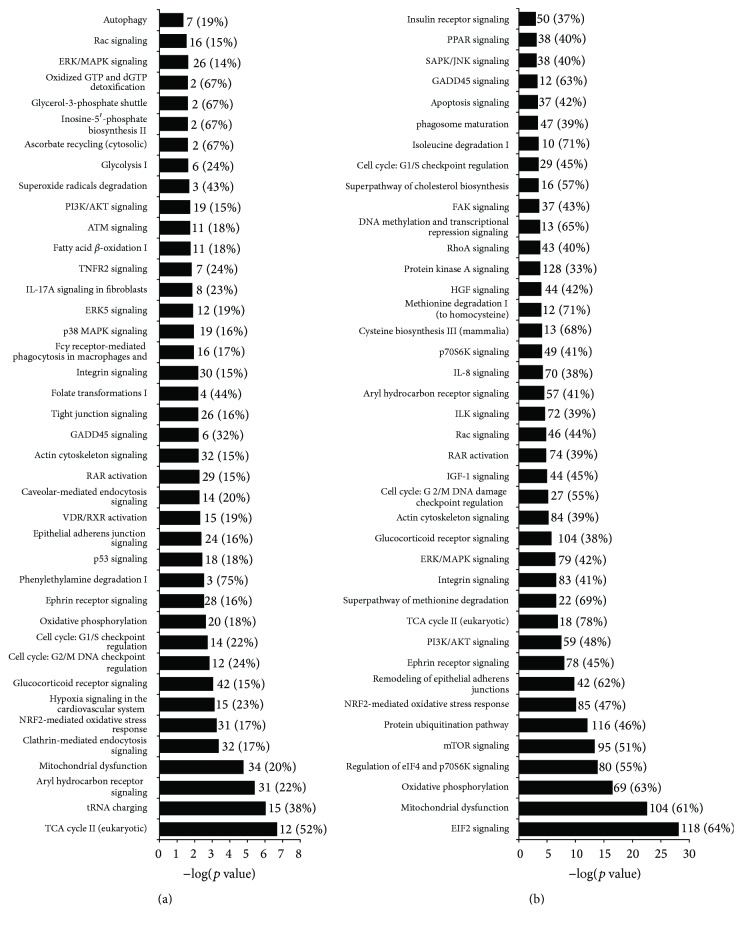
IPA analysis of differentially expressed host genes in VACV-infected cells. The list of SDEGs identified after VACV infection was used in a pathway enrichment analysis with IPA software. The 40 most significant pathways identified at 4 hpi (a) and 9 hpi (b) after VACV infection are shown. The *x*-axis represents the *p* value, indicating the significance of enrichment for the corresponding gene set. The values are plotted in a negative log_10_ scale.

By contrast, at 4 hpi with replication competent VACV, around 30% of total reads matched the virus genome, and a total of 2228 cellular genes were significantly differentially expressed compared to uninfected cells, 887 upregulated and 1341 downregulated (Table 1). In order to gain a comprehensive understanding of the transcriptomic changes induced by VACV infection we evaluated pathway enrichment by these SDEGs using Ingenuity Pathway Analysis (IPA) software. At this time postinfection the analysis identified severe alterations in cellular energy metabolism, since tricarboxylic acid (TCA) cycle, mitochondrial dysfunction, glycolysis, or oxidative phosphorylation represented the most significantly enriched pathways (Figure 3(a)). Some of these pathways were predicted to be inhibited, indicating that infection was suppressing levels of a broad variety of proteins involved in energy metabolism early in infection. Signalling related to cell proliferation and differentiation was also found to be clearly affected during VACV infection, and examples were ERK/MAPK or PI3K/AKT signalling pathways that were modified. Differential expression of pathways specifically associated with cell-cycle arrest, such as G1/S and G2/M DNA damage checkpoints, p53 signalling, or ATM signalling, were also enriched following infection. Other significantly enriched pathways, such as actin signalling, Rac signalling, and integrin signalling, were related to cell migration and are consistent with the previously described VACV-induced cell motility during infection [22].

Most of these enriched pathways altered at 4 hpi were also found to be modified later in infection (at 9 hpi, Figure 3(b)), showing higher *p* values. However, at 9 hpi the analysis detected a striking overrepresentation of cellular genes involved in the modulation of protein translation. We observed the downregulation of 165 genes encoding ribosomal proteins and 45 encoding translation initiation factors. As a consequence, the most significantly enriched pathways identified at this time postinfection included EIF2 signalling, and regulation of EIF4 and p7056K and mTOR signalling (Figure 3).

### 3.3. The Absence of B18 during VACV Infection Does Not Markedly Alter the Cellular Gene Expression Profile

**Figure 4:**
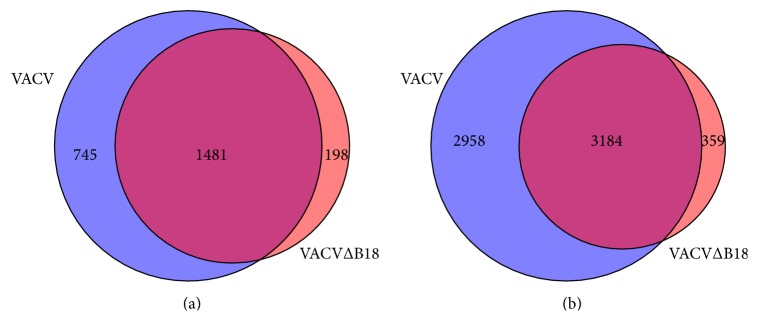
Effect of B18 absence on host gene expression during VACV infection. Venn diagrams showing the number of overlapped transcripts corresponding to cellular genes differentially expressed between VACV- and VACVΔB18-infected cells at 4 hpi (a) and 9 hpi (b) are displayed.

Previous analysis revealed the absence of IFN related pathways among enriched pathways altered after VACV infection, suggesting the viral downmodulation of type I IFN based host responses. Consistent with this, some of the ISGs with previously known antiviral activity, such as APOL9A, APOL9B, OAS1a, or OAS1g, exhibited lower expression levels in VACV-infected cells at 4 hpi as compared to mock-infected cells. We next evaluated the impact of B18 absence on cell host response during infection by using a VACV deletion mutant lacking expression (VACVΔB18). L929 cells were infected with VACVΔB18 and the gene expression profile was determined at 4 and 9 hpi and then compared to mock-infected cells. As shown in Table 1, 20% and 60% of the total sequencing reads corresponded to viral genes at 4 and 9 hpi, respectively. At 4 h after VACVΔB18 infection, a total of 1973 cellular SDEGs were identified when compared to mock-infected cells and the corresponding pathway enrichment analysis with these genes revealed that although 188 SDEGs were found exclusively differentially expressed during VACVΔB18 infection, most of these changes in gene expression were similar to those induced at the same times during wild type VACV infection, and no additional pathways were found among the 200 most significant enriched pathways (Figure 4).

### 3.4. Inhibition of the ISG Signature during VACV Infection Is Not Exclusively Dependent on B18

**Figure 5:**
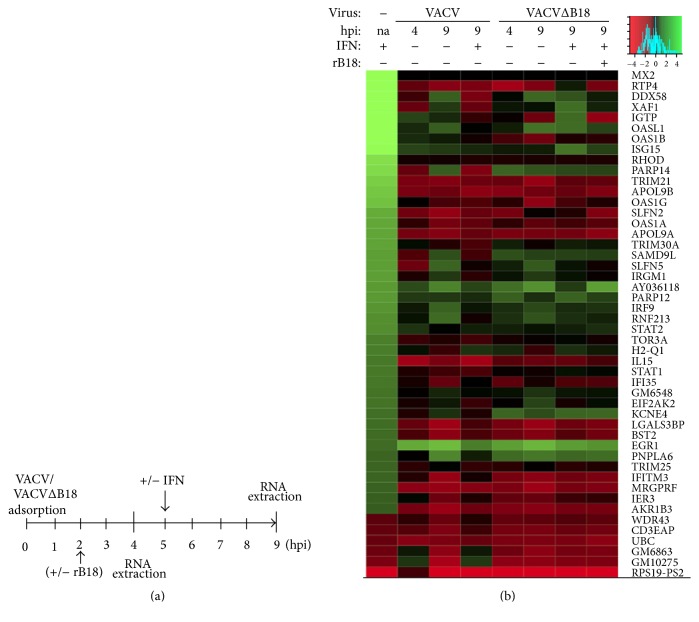
ISGs expression during VACV infection. (a) Diagram showing the experimental conditions to obtain RNA samples from infected cells. (b) Heatmap shows the expression levels of the ISGs previously identified relative to untreated cells, where indicated recombinant B18 protein (rB18) and/or mouse IFN*α* were/was added to cells at 2 and 5 hpi, respectively.

We also analyzed the IFN-mediated innate immune response after IFN treatment of infected cells. To this end, the expression levels of the ISGs previously identified after IFN treatment were determined by RNA-seq under various conditions. L929 cells were either (i) infected with wild type VACV and treated or not with IFN at 5 hpi, once the IFN inhibitor B18 had been produced and secreted; (ii) infected with VACVΔB18 and then treated or not with IFN (in the absence of B18); or (iii) infected with VACVΔB18 and supplemented with recombinant B18 before IFN addition. In all cases, total RNA was isolated at 9 hpi (4 h after IFN addition) and processed as indicated before. The results showed that addition of IFN to VACV-infected cells did not result in a clear activation pattern of the ISGs analyzed. We did not observe any difference in the ISG profile in cells infected with wild type VACV in the absence or presence of IFN, most likely due to the blocking action of secreted B18 to prevent IFN engagement with IFN cellular receptors. Surprisingly, even in cells infected with VACVΔB18, not producing B18, the addition of IFN did not result in an evident IFN based response, and the expression levels of the ISGs analyzed were similar to those found in VACVΔB18-infected cells in the presence of recombinant B18 protein and wild type VACV-infected cells (Figure 5).

**Figure 6:**
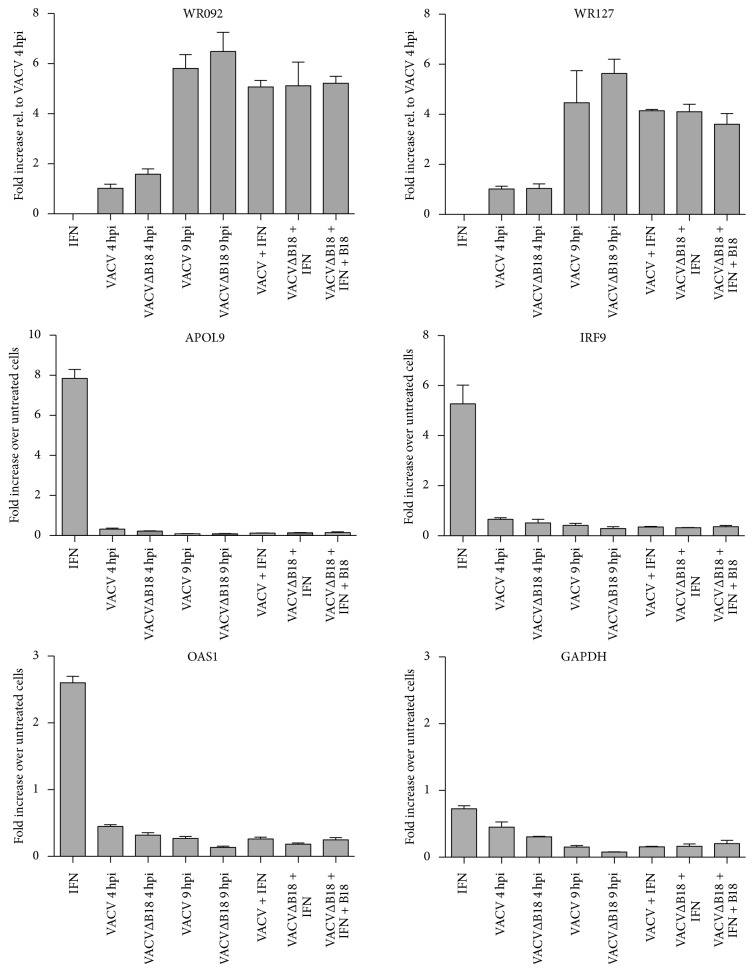
Confirmation of RNA-seq data by RT-qPCR during VACV infection. Gene expression for the indicated genes was assessed by RT-qPCR from VACV- or VACVΔB18-infected L929 cells at 4 hpi and 9 hpi. IFN was added to infected cells at 5 hpi where indicated. Expression levels of WR092 and WR127 VACV genes were also determined to monitor the progress of infection and relativized to VACV 4 hpi expression values. Mean from 3 biological replicates ± SEM and significant differences are displayed.

In an independent assay, with additional RNA samples, we could confirm these results by RT-qPCR in cells infected with wild type VACV or VACVΔB18 in the same conditions described above. We first verified the expression of the viral genes WR092 and WR127 in all infected cultures and observed increasing expression values from 4 to 9 hpi, independently of the addition of IFN. On the contrary, but in concordance with results from the RNA-seq, the expression values of the ISGs determined by RT-qPCR (APOL9, IRF9, and OAS1a) during wild type VACV or VACVΔB18 infections, and independently of the addition of IFN, were similar in all cases to those detected in nontreated cells (Figure 6). Finally, no significant modification of cellular GAPDH expression levels, determined by RT-qPCR, was observed at 4 h after infection while a slight decrease was observed at 9 h during wild type VACV or VACVΔB18 infection in either the absence or presence of IFN.

## 4. Discussion

The secreted type I IFN binding protein B18 from VACV represents a unique strategy employed by poxviruses to evade the host IFN response. Its important contribution to the virulence of VACV and ectromelia virus, a related mouse-specific virus that also encodes a B18 orthologue, has been demonstrated in mouse models of infection [13, 23]. This anti-IFN activity has also been identified in the highly virulent variola virus and monkeypox virus [17]. In this report we have addressed the ability of the secreted type I IFN binding protein to modulate the expression of host genes regulated by IFN, using an RNA-seq approach to monitor the global expression of host and viral genes.

First, we evaluated the impact of type I IFN on the gene expression profile, required to induce an antiviral state and protect cells from infection. In the case of L929 mouse fibroblasts, we found the expression of 46 genes affected by the addition of IFN-*α*. Consistent with previous results demonstrating the ability of B18 to block IFN effects [13–16], the modulation of host gene expression by IFN could be efficiently prevented by the action of B18.

Using the same RNA sequencing approach, the incubation of cells with purified B18 protein did not cause any significant change in gene expression, suggesting that no cell signalling is triggered by B18. This result is of particular relevance since, after secretion from infected cells, B18 interacts with GAGs at the surface of infected and adjacent uninfected cells [14, 16], and GAGs have been shown to regulate multiple signalling pathways. This is the case of some growth factors, such as fibroblast, hepatocyte, or vascular endothelial growth factors [24–26], where the participation of GAGs is essential for receptor-ligand engagement and the resulting signalling. In contrast, our results clearly indicate that the interaction of B18 with GAGs at the surface of L929 cells does not trigger any detectable cell signalling leading to changes in host gene expression. Consistent with our results, it has been reported that addition of purified recombinant B18 to primary mouse plasmacytoid cells does not induce type I IFN production, whereas these cells were able to produce type I IFN in response to TLR ligands [27].

In this report we have determined the cellular transcriptome profile to investigate the changes in host expression during VACV infection. The host reaction to VACV seems to start immediately after infection, as deduced from the set of genes differentially expressed 4 h after infection with UV-inactivated VACV. Among these, we found some NF-*κ*B regulated genes such as the proinflammatory chemokine RANTES/CCL5 gene, genes involved in the regulation of MAPK activity or the downregulation of antigen presentation related gene H2Q1, among others. It was somehow surprising that we did not detect more transcriptional activation in cells incubated with UV-inactivated virus, which should be able to attach and enter the cell. This may suggest that PRRs that activate cells in response to VACV infection detect mainly the viral genome that is being transcribed or replicated, rather than the small amount of virus particles that enter initially the cell (with viral proteins and DNA). Also, the incoming virus particle contains VH1, a phosphatase known to inhibit STAT1 and STAT2 activation, and may also prevent IFN responses in the absence of virus replication, as it was initially described [8]. Previous reports described the ability of inactivated VACV WR and monkeypox virus to induce the synthesis of IFN or IFN-inducible genes in plasmacytoid dendritic cells and macrophages, respectively [27, 28], but we could not detect the activation of these genes in L929 cells. Two factors could contribute to explaining these differences: the earlier timepoints analyzed compared with the previous studies or the fact that the levels of IFN production are cell type dependent. Plasmacytoid dendritic cells are considered to be the professional type I IFN producing cells after viral infections [29, 30] and secrete much more IFN-*α* than other cell types.

By contrast, a drastic change in the host gene expression profile occurred after 4 h of infection with replication competent VACV, mainly affecting biological functions related to metabolism, cell death and survival, cell development and proliferation, and cell movement. Previous studies with HeLa cells using microarrays or deep RNA sequencing showed a general decrease in the cellular mRNAs upon VACV infection [21, 31]. In our study, as previously reported by Yang et al. [21], we found that the proportion of VACV mRNA was approximately 30% of the total mRNA, and more than 50% of modified cellular genes were downregulated at 4 hpi, which is indicative of the virus-induced degradation of cellular mRNA that precedes host translation shutoff [32]. This effect was even more pronounced at 9 hpi. Since the aim of our study was to analyze the modulation of type I IFN responses by B18, we selected the times postinfection that allowed the synthesis and secretion into the supernatant of an effective amount of the B18 protein, which is produced at early times of infection. Under these conditions, we focused on type I IFN responses upon VACV infection.

Our RNA-seq data from VACV-infected samples is in concordance with previous reports showing that, unlike the highly attenuated modified VACV Ankara (MVA) strain, virulent VACV WR infection of mice or dendritic cell cultures did not raise IFN-*α* responses. It is also known that the lack of a functional* B18R* gene and other IFN inhibitors in MVA allows the development of an IFN based host response during infection [33]. However, our data indicated that the infection with VACVΔB18, lacking expression of the secreted type I IFN inhibitor, equally failed to raise an effective IFN host response and VACVΔB18 infection proceeded similarly to VACV infection in L929 cells. This result corroborates the existence of additional viral mechanisms to inhibit the induction of type I IFN responses, as previously indicated by* Waibler and cols *during VACV infection of pDCs [27]. While B18 remains as the only identified secreted type I IFN inhibitor, during the last years diverse VACV genes have been shown to have a direct role in the inhibition of the IFN production or the inhibition of the IFN signal transduction that takes place after type I IFN binding to cellular IFNAR [2]. Our results showed the lack of a functional IFN response during VACV infection in the absence of B18, and even after the addition of exogenous IFN-*α*, indicating that the IFN signalling downstream of IFNAR is impaired after VACV infection. We speculate that the virion associated phosphatase VH1, which dephosphorylates STAT1 and STAT2 to block downstream IFN-*α* signalling [8, 9], may contribute, together with other VACV genes, to explaining this lack of IFN responses during VACV infection in the absence of B18 function. In the cellular experimental system used here all cells were initially infected with VACV and hence the inhibition of the IFN response by B18 cannot be appreciated. The effect of B18 on virus replication in cell cultures treated with IFN is evident under other circumstances, such as when IFN is added a few hours after infection, as was illustrated in a previous report [14]. Deletion of the type I IFN binding protein in the VACV strain NYVAC has been reported to trigger the activation of IRF3, IRF7, and STAT1 and to increase the production of ISGs in human monocytes, in a transcriptomic analysis using microarrays [34]. The reasons for the different results reported in the previous report may be due to a different response in human monocytes or to the use of a highly attenuated VACV strain lacking many immunomodulatory genes, such as* C4L*,* N1L,* or* N2L*, which have been implicated in the modulation of intracellular signalling events [35–37]. Also, the recombinant viruses used in the NYVAC transcriptional studies have not been controlled for the potential inadvertent selection of mutations during the generation of the recombinant viruses, through the construction of revertant viruses or sequencing of the complete viral genomes, and thus the presence of additional mutations in other genes that may influence the reported results cannot be formally ruled out [38, 39].

The contribution of the secreted type I IFN binding protein to virus virulence and immune evasion becomes evident in mouse models of VACV and ectromelia virus infection, where mutant viruses show an attenuated phenotype that is dramatic in the mousepox model [13, 23]. In the animal host, the expression of a secreted IFN inhibitor is relevant to efficiently block the protective effects of IFN, which is produced in response to infection and is able to trigger IFN-mediated antiviral activities in neighbouring cells and restrict virus spread [23].

## 5. Conclusion

We have used RNA-seq to study by the modulation of the type I IFN response by VACV and the secreted type I IFN binding protein B18. This analysis identified cellular pathways modulated during VACV infection or induced by UV-inactivated virus particles. VACV B18 was a potent inhibitor of the type I IFN response, consistent with its ability to bind with high affinity IFN and to prevent its interaction with cellular IFNAR. VACVΔB18 inhibits the IFN response to an extent similar to that of wild type VACV, indicating that VACV encodes numerous mechanisms to block the IFN response and that the contribution of B18 to immune evasion is more evident in infected mice than in tissue culture. We also show that the interaction of B18 with cell surface GAGs does not trigger a specific host response leading to changes in host gene expression. The RNA-seq methodology allows the evaluation of the global gene expression in infected cells and the modulation of IFN responses by the VACV type I IFN binding protein. Future RNA-seq studies in VACV-infected mice may dissect better the ability of B18 to modulate the type I IFN-mediated response in different tissues.

## Supplementary Material

Supplementary material includes the sequence of the primers used for RT-PCR, and Tables containing the significant differentially expressed genes (SDEGs) and their expression values.

## Figures and Tables

**Table 1:** Alignment of Illumina reads and number of differentially expressed genes from infected cells with the indicated viruses.

	PLWUV VACV		VACV 4 hpi		VACV 9 hpi		VACVΔB18 4 hpi		VACVΔB18 9 hpi	
Total reads aligned^a^	150.810.958		117.443.318		181.794.658		139.017.860		155.220.273	
Viral reads^b^	260.091 (0.17%)		37.708.314 (32.1%)		144.727.059 (79.6%)		25.497.885 (18.4%)		90.326.955 (58.2%)	

Cellular SDEGs	Up	Down	Up	Down	Up	Down	Up	Down	Up	Down

	18	24	887	1341	2398	3753	660	1013	1238	2309

^a^Aligned to either mouse or VACV Western Reserve genomes.

^b^Aligned exclusively to VACV Western Reserve genome.

## References

[1] Haller O., Kochs G., Weber F. (2006). The interferon response circuit: induction and suppression by pathogenic viruses. *Virology*.

[2] Smith G. L., Benfield C. T. O., Maluquer de Motes C. (2013). Vaccinia virus immune evasion: mechanisms, virulence and immunogenicity. *Journal of General Virology*.

[3] Versteeg G. A., García-Sastre A. (2010). Viral tricks to grid-lock the type I interferon system. *Current Opinion in Microbiology*.

[4] Schneider W. M., Chevillotte M. D., Rice C. M. (2014). Interferon-stimulated genes: a complex web of host defenses. *Annual Review of Immunology*.

[5] Alcami A. (2003). Viral mimicry of cytokines, chemokines and their receptors. *Nature Reviews Immunology*.

[6] Perdiguero B., Esteban M. (2009). The interferon system and vaccinia virus evasion mechanisms. *Journal of Interferon and Cytokine Research*.

[7] Bowie A., Kiss-Toth E., Symons J. A., Smith G. L., Dower S. K., O'Neill L. A. J. (2000). A46R and A52R from vaccinia virus are antagonists of host IL-1 and toll-like receptor signaling. *Proceedings of the National Academy of Sciences of the United States of America*.

[8] Najarro P., Traktman P., Lewis J. A. (2001). Vaccinia virus blocks gamma interferon signal transduction: viral VH1 phosphatase reverses Stat1 activation. *Journal of Virology*.

[9] Mann B. A., Huang J. H., Li P. (2008). Vaccinia virus blocks Stat1-dependent and Stat1-independent gene expression induced by type I and type II interferons. *Journal of Interferon and Cytokine Research*.

[10] Carroll K., Elroy-Stein O., Moss B., Jagus R. (1993). Recombinant vaccinia virus K3L gene product prevents activation of double-stranded RNA-dependent, initiation factor 2*α*-specific protein kinase. *Journal of Biological Chemistry*.

[11] Chang H.-W., Jacobs B. L. (1993). Identification of a conserved motif that is necessary for binding of the vaccinia virus E3L gene products to double-stranded RNA. *Virology*.

[12] Guerra S., Cáceres A., Knobeloch K.-P., Horak I., Esteban M. (2008). Vaccinia virus E3 protein prevents the antiviral action of ISG15. *PLoS Pathogens*.

[13] Symons J. A., Alcamí A., Smith G. L. (1995). Vaccinia virus encodes a soluble type I interferon receptor of novel structure and broad species soecificity. *Cell*.

[14] Alcamí A., Symons J. A., Smith G. L. (2000). The vaccinia virus soluble alpha/beta interferon (IFN) receptor binds to the cell surface and protects cells from the antiviral effects of IFN. *Journal of Virology*.

[15] Colamonici O. R., Domanski P., Sweitzer S. M., Larner A., Buller R. M. L. (1995). Vaccinia virus B18R gene encodes a type I interferon-binding protein that blocks interferon *α* transmembrane signaling. *Journal of Biological Chemistry*.

[16] Montanuy I., Alejo A., Alcami A. (2011). Glycosaminoglycans mediate retention of the poxvirus type I interferon binding protein at the cell surface to locally block interferon antiviral responses. *FASEB Journal*.

[17] Fernandez de Marco Mdel M., Alejo A., Hudson P., Damon I. K., Alcami A. (2010). The highly virulent variola and monkeypox viruses express secreted inhibitors of type I interferon. *The FASEB Journal*.

[18] Tsung K., Yim J. H., Marti W., Buller R. M. L., Norton J. A. (1996). Gene expression and cytopathic effect of vaccinia virus inactivated by psoralen and long-wave UV light. *Journal of Virology*.

[19] Trapnell C., Roberts A., Goff L. (2012). Differential gene and transcript expression analysis of RNA-seq experiments with TopHat and Cufflinks. *Nature Protocols*.

[20] Livak K. J., Schmittgen T. D. (2001). Analysis of relative gene expression data using real-time quantitative PCR and the 2-ΔΔCT method. *Methods*.

[21] Yang Z., Bruno D. P., Martens C. A., Porcella S. F., Moss B. (2010). Simultaneous high-resolution analysis of vaccinia virus and host cell transcriptomes by deep RNA sequencing. *Proceedings of the National Academy of Sciences of the United States of America*.

[22] Sanderson C. M., Hollinshead M., Smith G. L. (2000). The vaccinia virus A27L protein is needed for the microtubule-dependent transport of intracellular mature virus particles. *Journal of General Virology*.

[23] Xu R.-H., Cohen M., Tang Y. (2008). The orthopoxvirus type I IFN binding protein is essential for virulence and an effective target for vaccination. *Journal of Experimental Medicine*.

[24] Ashikari-Hada S., Habuchi H., Kariya Y., Kimata K. (2005). Heparin regulates vascular endothelial growth factor165-dependent mitogenic activity, tube formation, and its receptor phosphorylation of human endothelial cells. Comparison of the effects of heparin and modified heparins. *The Journal of Biological Chemistry*.

[25] McDowell L. M., Frazier B. A., Studelska D. R. (2006). Inhibition or activation of apert syndrome FGFR2 (S252W) signaling by specific glycosaminoglycans. *Journal of Biological Chemistry*.

[26] Zioncheck T. F., Richardson L., Liu J. (1995). Sulfated oligosaccharides promote hepatocyte growth factor association and govern its mitogenic activity. *Journal of Biological Chemistry*.

[27] Waibler Z., Anzaghe M., Frenz T. (2009). Vaccinia virus-mediated inhibition of type I interferon responses is a multifactorial process involving the soluble type I interferon receptor B18 and intracellular components. *Journal of Virology*.

[28] Rubins K. H., Hensley L. E., Relman D. A., Brown P. O. (2011). Stunned silence: gene expression programs in human cells infected with monkeypox or vaccinia virus. *PLoS ONE*.

[29] Colonna M., Krug A., Cella M. (2002). Interferon-producing cells: on the front line in immune responses against pathogens. *Current Opinion in Immunology*.

[30] Liu Y.-J. (2005). IPC: professional type 1 interferon-producing cells and plasmacytoid dendritic cell precursors. *Annual Review of Immunology*.

[31] Guerra S., López-Fernández L. A., Pascual-Montano A., Muñoz M., Harshman K., Esteban M. (2003). Cellular gene expression survey of vaccinia virus infection of human HeLa cells. *Journal of Virology*.

[32] Rice A. P., Roberts B. E. (1983). Vaccinia virus induces cellular mRNA degradation. *Journal of Virology*.

[33] Waibler Z., Anzaghe M., Ludwig H. (2007). Modified vaccinia virus Ankara induces toll-like receptor-independent type I interferon responses. *Journal of Virology*.

[34] Delaloye J., Filali-Mouhim A., Cameron M. J. (2015). Interleukin-1- and type I interferon-dependent enhanced immunogenicity of an NYVAC-HIV-1 Env-Gag-Pol-Nef vaccine vector with dual deletions of type I and Type II interferon-binding proteins. *Journal of Virology*.

[35] DiPerna G., Stack J., Bowie A. G. (2004). Poxvirus protein N1L targets the I-*κ*B kinase complex, inhibits signaling to NF-*κ*B by the tumor necrosis factor superfamily of receptors, and inhibits NF-*κ*B and IRF3 signaling by toll-like receptors. *Journal of Biological Chemistry*.

[36] Ember S. W. J., Ren H., Ferguson B. J., Smith G. L. (2012). Vaccinia virus protein C4 inhibits NF-*κ*B activation and promotes virus virulence. *Journal of General Virology*.

[37] Ferguson B. J., Benfield C. T. O., Ren H. (2013). Vaccinia virus protein N2 is a nuclear IRF3 inhibitor that promotes virulence. *Journal of General Virology*.

[38] Gómez C. E., Perdiguero B., Nájera J. L. (2012). Removal of vaccinia virus genes that block interferon type I and II pathways improves adaptive and memory responses of the HIV/AIDS vaccine candidate NYVAC-C in mice. *Journal of Virology*.

[39] Kibler K. V., Gomez C. E., Perdiguero B. (2011). Improved NYVAC-based vaccine vectors. *PLoS ONE*.

