# Supplementary material for: RNA-Seq Based Transcriptome Analysis of the Type I Interferon Host Response upon Vaccinia Virus Infection of Mouse Cells
Source: J Immunol Res. 2017 Feb 9;2017:5157626. doi: 10.1155/2017/5157626 (PMC5322442; doi:10.1155/2017/5157626)
Supplement: Supplementary file 1 — Supplementary material includes the sequence of the primers used for RT-PCR, and Tables containing the significant differentially expressed genes (SDEGs) and their expression values. [file 5157626.f1.pdf]

Table S1. Primers sequence used in qPCR analyses.

Gene	Gene description	Forward (5´- 3´)	Reverse (5´- 3´)
**APOL9a**	Apolipoprotein 9a	cagtggatacctgtatagcctgg	tctgcctctgtctgctatgg
**b-ACT**	b-actin	ctaaggccaaccgtgaaaag	accagaggcatacagggaca
**DBF4**	DBF4-type zinc finger-containing protein 1	tatggacggcgataaatgtg	tcgagatcttcgtatttctgtaagc
**L5R**	Vaccinia virus WR092 gene	ccatggatgacaactcaaaca	tcatctgcgaagaacatcgt
**A8R**	Vaccinia virus WR127 gene	gatgcgttgacgctgtaaaa	tgcatccttttgatgcgata
**GAPDH**	Glyceraldehyde-3-phosphate dehydrogenase	ctcccactcttccaccttcg	cataccaggaaatgagcttgacaa
**IRF9**	Interferon regulatory factor 9	cagcaactgcaactctgagc	actcggccaccatagatgaa
**MRPL2**	Mitochondrial ribosomal protein L2	gagcaatgtcctccttcagc	tcttccaggacacaaacttgg
**OAS1a**	2'-5'-oligoadenylate synthetase 1	agcggaacttcctgaagcag	caggcaaagacagtgagcaa

Table S2. Significant differentially expressed genes after IFN induction.

Gene symbol	Mock FPKM	IFN FPKM	log2(FC)	p-value	Description
Mx2	0	1,42489	Inf	5,00E-05	myxovirus (influenza virus) resistance 2 [Source:MGI Symbol;Acc:MGI:97244]
Rtp4	0,224564	19,468	6,44	5,00E-05	receptor transporter protein 4 [Source:MGI Symbol;Acc:MGI:1915025]
Ddx58	0,3487	20,9985	5,91	5,00E-05	DEAD (Asp-Glu-Ala-Asp) box polypeptide 58 [Source:MGI Symbol;Acc:MGI:2442858]
Xaf1	0,221216	11,3076	5,68	5,00E-05	XIAP associated factor 1 [Source:MGI Symbol;Acc:MGI:3772572]
Igtp	0,119031	4,99169	5,39	0,00015	interferon gamma induced GTPase [Source:MGI Symbol;Acc:MGI:107729]
Oasl1	0,317158	13,1148	5,37	5,00E-05	2'-5' oligoadenylate synthetase-like 1 [Source:MGI Symbol;Acc:MGI:2180849]
Oas1b	0,139622	4,96127	5,15	5,00E-05	2'-5' oligoadenylate synthetase 1B [Source:MGI Symbol;Acc:MGI:97430]
Isg15	0,578739	17,5302	4,92	5,00E-05	ISG15 ubiquitin-like modifier [Source:MGI Symbol;Acc:MGI:1855694]
Rhod	18,0381	345,836	4,26	5,00E-05	ras homolog gene family, member D [Source:MGI Symbol;Acc:MGI:108446]
Parp14	0,0977489	1,71159	4,13	5,00E-05	poly (ADP-ribose) polymerase family, member 14 [Source:MGI Symbol;Acc:MGI:1919489]
Trim21	0,430314	6,05298	3,81	5,00E-05	tripartite motif-containing 21 [Source:MGI Symbol;Acc:MGI:106657]
Apol9a	2,08982	26,6401	3,67	5,00E-05	apolipoprotein L 9a [Source:MGI Symbol;Acc:MGI:3606001]
Oas1g	0,776779	8,88173	3,52	5,00E-05	2'-5' oligoadenylate synthetase 1G [Source:MGI Symbol;Acc:MGI:97429]
Helz2	2,50527	23,8868	3,25	5,00E-05	helicase with zinc finger 2, transcriptional coactivator [Source:MGI Symbol;Acc:MGI:2385169]
Slfn2	0,252734	1,98815	2,98	0,00015	schlafen 2 [Source:MGI Symbol;Acc:MGI:1313258]
Oas1a	1,41434	10,2379	2,86	5,00E-05	2'-5' oligoadenylate synthetase 1A [Source:MGI Symbol;Acc:MGI:2180860]
Apol9b	2,66209	18,551	2,80	5,00E-05	apolipoprotein L 9b [Source:MGI Symbol;Acc:MGI:1919148]
Trim30a	1,07502	7,16009	2,74	5,00E-05	tripartite motif-containing 30A [Source:MGI Symbol;Acc:MGI:98178]
Samd9l	1,47019	9,24891	2,65	5,00E-05	sterile alpha motif domain containing 9-like [Source:MGI Symbol;Acc:MGI:1343184]
Slfn5	0,36026	2,24497	2,64	5,00E-05	schlafen 5 [Source:MGI Symbol;Acc:MGI:1329004]
Irgm1	7,42535	46,0772	2,63	5,00E-05	immunity-related GTPase family M member 1 [Source:MGI Symbol;Acc:MGI:107567]
Parp12	0,493812	2,73206	2,47	5,00E-05	poly (ADP-ribose) polymerase family, member 12 [Source:MGI Symbol;Acc:MGI:2143990]
Irf9	6,37807	33,2275	2,38	5,00E-05	interferon regulatory factor 9 [Source:MGI Symbol;Acc:MGI:107587]
Rnf213	5,66188	26,3644	2,22	5,00E-05	ring finger protein 213 [Source:MGI Symbol;Acc:MGI:1289196]
Stat2	2,5659	11,7023	2,19	5,00E-05	signal transducer and activator of transcription 2 [Source:MGI Symbol;Acc:MGI:103039]
Tor3a	10,1315	36,1723	1,84	5,00E-05	torsin family 3, member A [Source:MGI Symbol;Acc:MGI:1353652]
H2-Q1	3,41199	11,8566	1,80	5,00E-05	histocompatibility 2, Q region locus 1 [Source:MGI Symbol;Acc:MGI:95928]
Il15	2,12905	6,78449	1,67	5,00E-05	interleukin 15 [Source:MGI Symbol;Acc:MGI:103014]
Stat1	2,63919	8,31407	1,66	5,00E-05	signal transducer and activator of transcription 1 [Source:MGI Symbol;Acc:MGI:103063]
Ifi35	3,66682	11,2891	1,62	5,00E-05	interferon-induced protein 35 [Source:MGI Symbol;Acc:MGI:1917360]
Eif2ak2	7,79477	22,6396	1,54	5,00E-05	eukaryotic translation initiation factor 2-alpha kinase 2 [Source:MGI Symbol;Acc:MGI:1353449]
Kcne4	3,37498	8,97622	1,41	5,00E-05	potassium voltage-gated channel, Isk-related subfamily, gene 4 [Source:MGI Symbol;Acc:MGI:1891125]
Lgals3bp	31,7365	81,3106	1,36	5,00E-05	lectin, galactoside-binding, soluble, 3 binding protein [Source:MGI Symbol;Acc:MGI:99554]
Bst2	12,9175	32,3267	1,32	5,00E-05	bone marrow stromal cell antigen 2 [Source:MGI Symbol;Acc:MGI:1916800]
Egr1	3,44869	8,61428	1,32	5,00E-05	early growth response 1 [Source:MGI Symbol;Acc:MGI:95295]
Ifi27	41,3458	102,436	1,31	5,00E-05	interferon, alpha-inducible protein 27 [Source:MGI Symbol;Acc:MGI:1277180]
Pnpla6	6,39246	14,3499	1,17	5,00E-05	patatin-like phospholipase domain containing 6 [Source:MGI Symbol;Acc:MGI:1354723]
Trim25	13,252	29,4216	1,15	5,00E-05	tripartite motif-containing 25 [Source:MGI Symbol;Acc:MGI:102749]
Ifitm3	168,338	362,716	1,11	5,00E-05	interferon induced transmembrane protein 3 [Source:MGI Symbol;Acc:MGI:1913391]
Mrgprf	12,0076	25,4772	1,09	5,00E-05	MAS-related GPR, member F [Source:MGI Symbol;Acc:MGI:2384823]
Ier3	50,7915	102,292	1,01	5,00E-05	immediate early response 3 [Source:MGI Symbol;Acc:MGI:104814]
Akr1b3	351,422	700,47	1,00	5,00E-05	aldo-keto reductase family 1, member B3 (aldose reductase) [Source:MGI Symbol;Acc:MGI:1353494]
Wdr43	93,5366	49,7774	-0,91	1,00E-04	WD repeat domain 43 [Source:MGI Symbol;Acc:MGI:1919765]
Cd3eap	35,5701	17,7292	-1,00	0,00015	CD3E antigen, epsilon polypeptide associated protein [Source:MGI Symbol;Acc:MGI:1917583]
Ubc	299,551	138,413	-1,11	5,00E-05	ubiquitin C [Source:MGI Symbol;Acc:MGI:98889]
Rps19-ps2	5,09322	0	-Inf	5,00E-05	ribosomal protein S19, pseudogene 2 [Source:MGI Symbol;Acc:MGI:3647073]

Table S3. VACV gene expression after infection with UV-inactivated VACV.

VACV WR gene	FPKM (1)	Temporal Expression (2)
**196**	448,453	E1.2
**089**	334,426	E1.1
**158**	257,419	E1.1
**053**	250,594	E1.1
**038**	228,898	E1.1
**059**	176,753	E1.1
184	154,072	E1.2
**160**	149,1	E1.1
**034**	117,086	E1.1
**083**	108,275	E1.2
**041**	107,406	E1.2
**032**	90,2349	E1.1
**112**	86,678	E1.2
**047**	84,7748	E1.2
**029**	82,5136	E1.1
**156**	63,8192	E1.1
**172**	63,774	E1.1
**169**	62,592	E1.2
**190**	60,2621	E1.1
**031**	57,8544	E1.2
**200**	56,199	E1.1
**154**	53,0669	E1.2
**050**	52,7462	E1.1
**188**	44,3952	E1.2
**094**	43,4401	E1.2
**022**	41,9533	E1.1
**072**	40,2754	E1.1
**046**	39,0076	E1.2
**039**	36,1997	E1.1
**021**	35,4091	E1.2
**103**	35,3938	E1.1
**194**	33,5508	E1.1
**193**	33,394	E1.2
**174**	33,2188	E1.1
**124**	32,9562	E1.2
**159**	29,7016	E1.1
**037**	28,6559	E1.1

VACV WR gene	FPKM (1)	Temporal Expression (2)
**043**	27,483	E1.2
**061**	26,18	E1.1
**173**	26,1105	E1.2
**054**	24,4903	E1.1
**191**	24,1053	PR
**201**	23,857	E1.2
127	23,538	E1.1
**170**	23,3367	E1.2
**195**	23,0083	E1.1
**165**	21,6519	E1.2
**117**	20,2527	E1.2
**028**	18,7823	E1.2
**023**	18,4036	E1.2
**152**	16,1524	E1.2
**109**	15,664	E1.2
**060**	15,6077	E1.1
**068**	15,4175	E1.1
**183**	15,0487	E1.2
**024**	13,6144	E1.2
**177**	13,4575	E1.1
**045**	13,4289	E1.2
**181**	12,5421	E1.2
**175**	12,2789	E1.2
**030**	12,2445	E1.2
**143**	12,0635	E1.2
**044**	11,964	E1.2
**202**	10,018	E1.2
**137**	9,63951	PR
**055**	8,78575	E1.2
**114**	8,30566	E1.1
**178**	8,12113	E1.2
**040**	7,61685	E1.2
**042**	7,22993	E1.2
**095**	7,20461	E1.2
**051**	7,12493	E1.2
**110**	6,78521	E1.2

(1) FPKM, Fragments Per Kilobase Of Exon Per Million Fragments Mapped.
(2) **Temporal expression according to *Yang Z., et al.*** E1.1=Early subclaster 1, E1.2=Early subclaster 2 and PR=Post replicative.

Table S4. Significant differentially expressed host genes after infection with inactivated VACV.

Gene symbol	Mock FPKM	PLWUV_VACV FPKM	log2(FC)	p-value	Description
Pou5f1	0	0,387441	Inf	4,00E-04	POU domain, class 5, transcription factor 1 [Source:MGI Symbol;Acc:MGI:101893]
Ccl5	0	0,525911	Inf	5,00E-05	chemokine (C-C motif) ligand 5 [Source:MGI Symbol;Acc:MGI:98262]
Fosb	1,358	7,22105	2,41073	5,00E-05	FBJ osteosarcoma oncogene B [Source:MGI Symbol;Acc:MGI:95575]
H3f3a-ps2	4,32726	21,1427	2,28864	3,00E-04	H3 histone, family 3A, pseudogene 2 [Source:MGI Symbol;Acc:MGI:1101758]
Rnaset2b	0,584778	2,37846	2,02407	3,00E-04	ribonuclease T2B [Source:MGI Symbol;Acc:MGI:3702087]
Pgk1-rs7	1,58989	6,32818	1,99286	5,00E-05	phosphoglycerate kinase-1, related sequence-7 [Source:MGI Symbol;Acc:MGI:97562]
Gm11808	44,2146	156,197	1,82077	5,00E-05	predicted gene 11808 [Source:MGI Symbol;Acc:MGI:3649356]
Apbb2	19,4623	67,1926	1,78762	1,00E-04	amyloid beta (A4) precursor protein-binding, family B, member 2 [Source:MGI Symbol;Acc:MGI:108405]
Gm13826	32,051	108,813	1,76341	2,00E-04	predicted gene 13826 [Source:MGI Symbol;Acc:MGI:3651519]
H2-Q1	3,37993	11,42	1,7565	5,00E-05	histocompatibility 2, Q region locus 1 [Source:MGI Symbol;Acc:MGI:95928]
Rps13-ps2	10,7971	33,9251	1,65171	5,00E-05	ribosomal protein S13, pseudogene 2 [Source:MGI Symbol;Acc:MGI:3704295]
Gm2000	13,7296	40,6802	1,56704	0,00015	predicted gene 2000 [Source:MGI Symbol;Acc:MGI:3780170]
Gm10288	21,5221	62,0438	1,52747	5,00E-05	predicted gene 10288 [Source:MGI Symbol;Acc:MGI:3704227]
Gm12918	22,5483	58,528	1,37611	1,00E-04	predicted gene 12918 [Source:MGI Symbol;Acc:MGI:3652005]
Tpt1-ps3	50,9163	120,877	1,24734	5,00E-05	tumor protein, translationally-controlled, pseudogene 3 [Source:MGI Symbol;Acc:MGI:2664997]
Rps23-ps	45,0603	105,712	1,23021	5,00E-05	ribosomal protein S23, pseudogene [Source:MGI Symbol;Acc:MGI:3705429]
Serpine1	97,491	228,655	1,22983	5,00E-05	serine (or cysteine) peptidase inhibitor, clade E, member 1 [Source:MGI Symbol;Acc:MGI:97608]
Rps6-ps4	31,1679	72,6403	1,22071	5,00E-05	ribosomal protein S6, pseudogene 4 [Source:MGI Symbol;Acc:MGI:3650907]
Dusp5	9,01692	20,1282	1,15851	5,00E-05	dual specificity phosphatase 5 [Source:MGI Symbol;Acc:MGI:2685183]
Spsb1	6,66087	13,6779	1,03806	0,00035	splA/ryanodine receptor domain and SOCS box containing 1 [Source:MGI Symbol;Acc:MGI:1921896]
Klf16	13,4366	26,9624	1,00478	0,00015	Kruppel-like factor 16 [Source:MGI Symbol;Acc:MGI:2153049]
Mid1	42,8261	84,6937	0,983766	2,00E-04	midline 1 [Source:MGI Symbol;Acc:MGI:1100537]
Hmox1	41,1287	75,07	0,868091	0,00055	heme oxygenase (decycling) 1 [Source:MGI Symbol;Acc:MGI:96163]
Mif	764,154	422,391	-0,855282	0,00025	macrophage migration inhibitory factor [Source:MGI Symbol;Acc:MGI:96982]
Sod3	59,645	31,9188	-0,901998	0,00015	superoxide dismutase 3, extracellular [Source:MGI Symbol;Acc:MGI:103181]
Cxcl1	46,1628	23,8182	-0,954665	0,00055	chemokine (C-X-C motif) ligand 1 [Source:MGI Symbol;Acc:MGI:108068]
Mt1	1080,16	546,469	-0,983029	5,00E-05	metallothionein 1 [Source:MGI Symbol;Acc:MGI:97171]
Rplp1	4572,05	2272,58	-1,00851	5,00E-05	ribosomal protein, large, P1 [Source:MGI Symbol;Acc:MGI:1927099]
Shfm1	109,21	52,556	-1,05517	5,00E-05	split hand/foot malformation (ectrodactyly) type 1 [Source:MGI Symbol;Acc:MGI:109238]
H2afj	16,3602	7,8444	-1,06045	5,00E-04	H2A histone family, member J [Source:MGI Symbol;Acc:MGI:3606192]
Akr1b3	348,17	163,409	-1,0913	5,00E-05	aldo-keto reductase family 1, member B3 (aldose reductase) [Source:MGI Symbol;Acc:MGI:1353494]
Atp5k	347,388	160,9	-1,11039	5,00E-05	ATP synthase, H+ transporting, mitochondrial F1F0 complex, subunit e [Source:MGI Symbol;Acc:MGI:106636]
Cdkn2d	32,7406	14,9265	-1,13321	1,00E-04	cyclin-dependent kinase inhibitor 2D (p19, inhibits CDK4) [Source:MGI Symbol;Acc:MGI:105387]
Tnfaip8	15,4048	6,72892	-1,19494	0,00015	tumor necrosis factor, alpha-induced protein 8 [Source:MGI Symbol;Acc:MGI:2147191]
Usp17la	6,86756	2,97834	-1,20529	2,00E-04	ubiquitin specific peptidase 17-like A [Source:MGI Symbol;Acc:MGI:107699]
Dab2ip	3,05626	1,30815	-1,22424	2,00E-04	disabled 2 interacting protein [Source:MGI Symbol;Acc:MGI:1916851]
Rplp2	1458,64	583,381	-1,32211	3,00E-04	ribosomal protein, large P2 [Source:MGI Symbol;Acc:MGI:1914436]
Rpl26	269,924	107,829	-1,32381	5,00E-05	ribosomal protein L26 [Source:MGI Symbol;Acc:MGI:106022]
Trib3	37,9265	15,0973	-1,32891	5,00E-05	tribbles homolog 3 (Drosophila) [Source:MGI Symbol;Acc:MGI:1345675]
Layn	8,11526	3,22519	-1,33125	5,00E-05	layilin [Source:MGI Symbol;Acc:MGI:2685357]
Gdf15	11,0194	4,33143	-1,34712	0,00055	growth differentiation factor 15 [Source:MGI Symbol;Acc:MGI:1346047]
Ddit4	7,93632	2,90396	-1,45045	2,00E-04	DNA-damage-inducible transcript 4 [Source:MGI Symbol;Acc:MGI:1921997]
Klf15	4,08803	1,3104	-1,6414	0,00045	Kruppel-like factor 15 [Source:MGI Symbol;Acc:MGI:1929988]
Chac1	19,1486	5,94093	-1,68848	5,00E-05	ChaC, cation transport regulator 1 [Source:MGI Symbol;Acc:MGI:1916315]
Gm5611	21,5466	6,399	-1,75154	5,00E-05	predicted gene 5611 [Source:MGI Symbol;Acc:MGI:3647121]
Phex	0,737842	0,202192	-1,86759	7,00E-04	phosphate regulating gene with homologies to endopeptidases on the X chromosome (hypophosphatemia, vitamin D resistant rickets) [Source:MGI Symbol;Acc:MGI:107489]
Sh2d3c	2,28625	0,618298	-1,88661	5,00E-05	SH2 domain containing 3C [Source:MGI Symbol;Acc:MGI:1351631]
Gm14323	5,46775	0,883133	-2,63024	7,00E-04	predicted gene 14323 [Source:MGI Symbol;Acc:MGI:3650742]
Abcg1	1,03044	0,160843	-2,67954	1,00E-04	ATP-binding cassette, sub-family G (WHITE), member 1 [Source:MGI Symbol;Acc:MGI:107704]
Gm14414	6,22852	0,943767	-2,72239	4,00E-04	predicted gene 14414 [Source:MGI Symbol;Acc:MGI:3652253]
Gm12033	24,1417	0,757299	-4,99452	5,00E-05	predicted gene 12033 [Source:MGI Symbol;Acc:MGI:3650772]
Rab42	0,294648	0	-Inf	7,00E-04	RAB42, member RAS oncogene family [Source:MGI Symbol;Acc:MGI:2441753]
Rps19-ps2	5,04479	0	-Inf	5,00E-05	ribosomal protein S19, pseudogene 2 [Source:MGI Symbol;Acc:MGI:3647073]
Rps19-ps1	5,04479	0	-Inf	5,00E-05	ribosomal protein S19, pseudogene 1 [Source:MGI Symbol;Acc:MGI:3782508]
